# Improving prevention and early diagnosis

**DOI:** 10.1186/1471-2334-14-S2-S6

**Published:** 2014-05-23

**Authors:** Virginie Supervie

**Affiliations:** 1 Sorbonne University, Pierre Louis Institute of Epidemiology and Public Health, Paris, France

## 

More than thirty years after the discovery of HIV, the HIV/AIDS epidemic continues to expand worldwide. Undiagnosed HIV infections and late diagnoses remain major public health challenges in all HIV-affected countries. According to recent estimates, the proportion of persons living with HIV unaware of their infection varies from >50% in sub-Saharan Africa to 20-50% in European countries and 20-30% in North America. In high-income countries, around 50% of new HIV diagnoses are made late (i.e., when CD4 count is <350 cells per μL); mean CD4 cell count at first presentation to medical care in high-income countries was slightly above 300 cells per μL in 2011, and has not increased significantly over the past 20 years. In low- and middle-income countries, most patients start HIV treatment with CD4 counts well below the recommended thresholds; the main reason for late initiation of HIV treatment is late diagnosis.

Undiagnosed HIV infections and late diagnoses have serious implications for both the individual and public health. Patients diagnosed late cannot benefit fully from timely initiation of HIV treatment and are more likely to experience HIV-related morbidity and premature mortality. Undiagnosed HIV-infected individuals have a higher risk of transmitting HIV than individuals aware of their HIV infection, because they are not engaged in HIV treatment (which greatly reduces the risk of transmission) and cannot make informed decisions about their sexual behavior. As a consequence, the majority of new HIV transmissions may originate from individuals who are unaware of their HIV infection.

Improving prevention and early diagnosis are thus key objectives to reduce mortality and turning the tide on HIV transmission. In this presentation, we will describe the non-biomedical HIV prevention methods, including early diagnosis, review and summarize the evidence supporting (or not) their effectiveness and discuss how these methods could be improved.

